# Selection of acute blood purification therapy according to severity score and blood lactic acid value in patients with septic shock

**DOI:** 10.4103/0972-5229.76080

**Published:** 2010

**Authors:** Yuichiro Sakamoto, Kunihiro Mashiko, Hisashi Matsumoto, Yoshiaki Hara, Noriyoshi Kutsukata, Hiroyuki Yokota

**Affiliations:** **From:** Department of Emergency and Critical Care Medicine, Chiba-Hokuso Hospital, Nippon Medical School, Japan

**Keywords:** Acute blood purification therapy, blood lactic acid, septic shock

## Abstract

**Aim:**

As an alternative method for acute blood purification therapy, continuous venovenous hemodiafiltration (CVVHDF) has been reported as an effective clinical treatment for critically ill patients, but the optimal column for performing CVVHDF remains controversial.

**Patients and Methods:**

We used direct hemoperfusion using a polymyxin B-immobilized fiber column (DHP-PMX) to treat 88 patients with septic shock. To determine the optimal acute blood purification therapy, we subsequently divided the patients into three groups: the first group underwent CVVHDF using a polymethylmethacrylate membrane hemofilter (PMMA) after undergoing DHP-PMX (28 cases), the second group underwent CVVHDF using a polyacrylonitrile membrane hemofilter (PAN) after undergoing DHP-PMX (26 cases), and the third group did not undergo CVVHDF after undergoing DHP-PMX (34 cases).

**Results:**

The overall survival rate was 54.5%, and patient outcome was significantly related to the Acute Physiology and Chronic Health Evaluation (APACHE) II score, the sepsis-related organ failure assessment (SOFA) score, and the blood lactic acid value before treatment (all P<0.0001). Only the PMMA-CVVHDF group showed a better outcome (survival rate of 78.6%) compared with the other groups (*P* = 0.0190). In addition, only the PMMA-CVVHDF group showed a significant improvement in the blood lactic acid level on day 3 (*P* = 0.0011).

**Conclusion:**

Our study suggests that the PMX column might be effective during the early phase of septic shock, before a high level of lactic acid is present. Furthermore, a PMMA column might be the most useful column for performing CVVHDF after DHP-PMX treatment, as suggested by the blood lactic acid value.

## Introduction

Sepsis, a systemic inflammatory response to infection, is a severe condition. Septic shock is associated with diffuse coagulopathy and multiple organ failure (MOF) and frequently leads to death. More research is needed to develop better directed therapies capable of reducing the high morbidity and mortality rates associated with sepsis.[1]

Direct hemoperfusion using a polymyxin B-immobilized fiber column (DHP-PMX) was first developed in Japan in 1994 and has since been used for the treatment of septic shock.[2]

As an alternative method for acute blood purification therapy, continuous venovenous hemodiafiltration (CVVHDF) has been reported as an effective treatment for critically ill patients, but the optimal column for performing CVVHDF remains controversial.

A recent paper reported that continuous hemodiafiltration using a polymethylmethacrylate membrane hemofilter (PMMA-CHDF) was effective for removing cytokines and enabled a wide range of clinical efficacy through cytokine modulation.[3] In the present study, we examined which column was optimal for performing CVVHDF in patients with septic shock.

## Patients and Methods

Eighty-eight septic patients who had undergone DHP-PMX were included in this study. Among these patients, DHP-PMX was performed within 12 hours after a diagnosis of sepsis was made. We included cases that underwent CVVHDF promptly after DHP-PMX treatment as well as cases that did not undergo CVVHDF for at least 36 hours after DHP-PMX treatment in the present study. These different treatment protocols were included to determine the best choice for acute blood purification therapy after DHP-PMX by comparing the survival rates, sepsis-related organ failure assessment (SOFA) scores,[4] and blood lactic acid values. The patients were divided into three groups: the first group underwent CVVHDF using a polymethylmethacrylate membrane hemofilter (PMMA) after undergoing DHP-PMX (28 cases), the second group underwent CVVHDF using a polyacrylonitrile membrane hemofilter (PAN) after undergoing DHP-PMX (26 cases), and the third group did not undergo CVVHDF after undergoing DHP-PMX (34 cases). The PAN-CVVHDF group was treated between January 2000 and March 2006, while the PMMA-CVVHDF group was treated between April 2006 and December 2008. Two periods were successive. The patients in the non-CVVHDF group were recruited during the PAN-CVVHDF group period, during which time CVVHDF was performed according to physician’s preference. All cases were not treated for collection of data and written consent was obtained. We did CVVHDF after DHP-PMX not only renal dysfunction but also septic shock.

Main indication of CVVHDF after DHP-PMX is that it removes inflammatory mediators.

The patients were divided into two groups according to the outcome (48 survivors, 40 deaths) to analyze the utility of the severity score for predicting outcome after DHP-PMX treatment. We also examined the relations between the severity scores such as the Acute Physiology and Chronic Health Evaluation (APACHE) II score,[5] the SOFA score, and the blood lactic acid value. The APACHE II score and the SOFA score were also evaluated and the blood lactic acid level was measured before DHP-PMX treatment.

Blood access for PMMA-CVVHDF and PAN-CVVHDF was established using a double-lumen catheter inserted into the femoral vein using the Seldinger method. Nafamostat mesilate (Torii Co., Ltd., Tokyo, Japan) was used for anticoagulation.

The approval of our institution’s ethics committee and informed consent from the patients were obtained. The results were expressed as the mean ± SD. Differences were analyzed using the Wilcoxon generalized test and the chi-squared test. The survival rate was analyzed using the Kaplan Meier method. A *P* value less than 0.05 was regarded as statistically significant.

## Results

DHP-PMX was performed in 88 patients (58 men and 30 women) of age between 20 and 87 years (mean 64.1 ± 13.7 years). The underlying diseases and the patient characteristics are shown in Table 1. The overall survival rate was about 54.5% at 90 days after onset.

**Table 1:** Patient characteristics before DHP-PMX

Characteristics	Data	
No. of patients	88	
Sex (male/female)	58/ 30	
Age (years), mean ± SE	64.1 ±13.7	
APACHE II score (mean ± SE)	24.0±9.2	
SOFA score (mean ± SE)	11.1±4.5	
Underlying diseases	Peritonitis	47cases
	Pneumonia	15cases
	Urinary Infection	5cases
	Others	21cases

**Figure 1:**
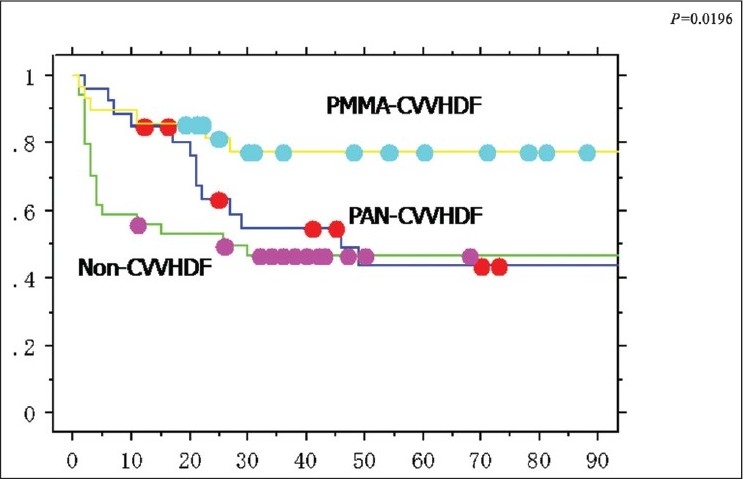
Significant differences between the APACHE II score and CVVHDF treatment were not observed relations between survival curve and types of apheresis therapy after DHP-PMX are shown. The outcome of the PMMA-CVVHDF group was significantly better than those of the PAN-CVVHDF group and the non-CVVHDF group (*P* = 0.0196)

Significant differences between the severity scores (APACHE II and SOFA scores) and CVVHDF treatment were not observed. The relation between the survival curve and the type of apheresis therapy after DHP-PMX is shown in Figure 1. The outcome of the PMMA-CVVHDF group was significantly better than that of the PAN-CVVHDF group or the non-CVVHDF group (*P* = 0.0196) [Figure 1].

**Figure 2:**
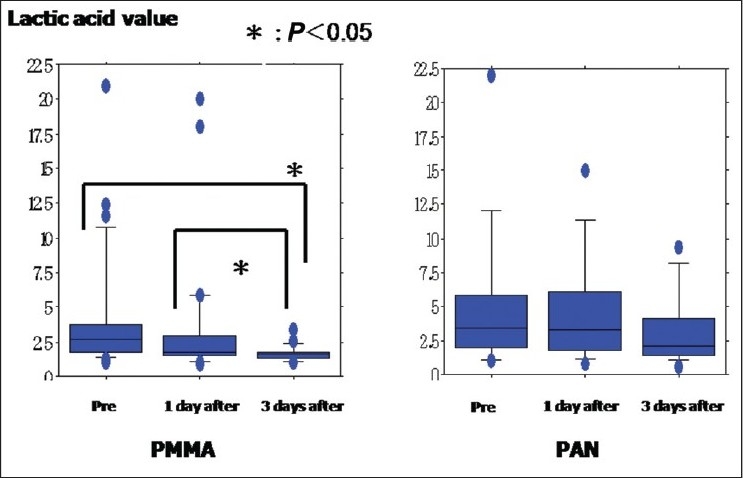
Significant differences between the SOFA score and CVVHDF treatment were not observed (a) Lactic acid levels after PMMA-CVVHDF: The lactic acid value at 3 days after PMMA-CVVHDF therapy was significantly better than the values obtained before and 1 day after this therapy (*P* = 0.0076 and *P* = 0.0444, respectively). (b) Lactic acid levels after PAN-CVVHDF: The lactic acid value after PAN-CVVHDF therapy was not significantly better than the values obtained before this therapy

**Figure 3:**
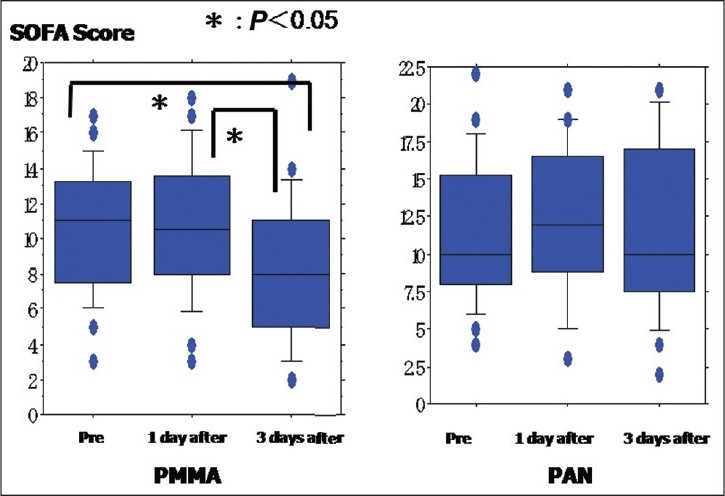
(a) SOFA scores after PMMA-CVVHDF: The SOFA score at 3 days after PMMA-CVVHDF therapy was significantly lower than those obtained before and 1 day after this therapy (*P* = 0.0102 and *P* = 0.0008, respectively). (b) SOFA scores after PAN-CVVHDF: The SOFA score after PAN-CVVHDF therapy was not significantly lower than those obtained before this therapy

The relationship between the lactic acid level and CVVHDF treatment is shown in Figure 2. The lactic acid level in the non-CVVHDF group was significantly higher than that in the PMMA group (*P* = 0.0315), but no significant difference was seen between the two CVVHDF treatment groups (PMMA group and PAN group) (data not shown). In the PMMA-CVVHDF group, the lactic acid value at 3 days after CVVHDF was significantly better than the values before and 1 day after CVVHDF [Figure 2]. In contrast, the lactic acid values obtained before and 1 and 3 days after treatment showed no significant improvement in the PAN-CVVHDF [Figure 2] and non-CVVHDF groups (data not shown). In the PMMA-CVVHDF group, the SOFA score obtained at 3 days after CVVHDF therapy was significantly lower than those obtained before and 1 day after [Figure 3]. However, the SOFA score at 3 days after therapy was not significantly different from those obtained before and 1 day after therapy in the PAN-CVVHDF [Figure 3] and non-CVVHDF groups (data not shown).

The relations between the severity scores and the outcomes of the septic cases are shown in Table 2. Before treatment, the APACHE II scores of the patients who survived and those who did not were 19.8 ± 7.8 and 28.7 ± 8.4, respectively. The SOFA scores of the patients who survived and those who did not were 8.7 ± 3.5 and 13.7 ± 3.9, respectively. The lactic acid values of the patients who survived and those who did not were 2.7 ± 1.6 and 7.4 ± 5.0, respectively (*P* < 0.0001) [Table 2]. Renal function before treatment and improved rate of renal function were not significantly different between PMMA-CVVHDF group and PAN-CVVHDF groups.

**Table 2:** Relationship between any scores and outcome in septic cases

Scores	Survived	Expired	*P* value
Number of cases	48	40	
Age	63.6±14.5	64.7±12.9	NS
APACHE II score	19.8±7.8	28.7±8.4	<0.0001
SOFA score	8.7±3.5	13.7±3.9	<0.0001
Lactic acid	2.7±1.6	7.4±5.0	<0.0001

## Discussion

Although intensive care has recently improved, sepsis remains a principal cause of MOF and is associated with high morbidity and mortality rates.[6] The results of a multicenter prospective randomized controlled trial for DHP-PMX have suggested that this treatment may improve cardiac and renal dysfunction caused by sepsis or septic shock,[7] and a recent systematic review of a pooled sample of 1390 patients showed that DHP-PMX therapy significantly lowered endotoxin levels, improved blood pressure, and reduced the mortality rate.[8] Therefore, this method is thought to be extremely useful for establishing new strategies for the treatment of septic patients.

Other therapies involving blood purification, including CVVHDF and continuous venovenous hemofiltration (CVVHF), have been reported to be effective for the treatment of sepsis.[9,10] Nevertheless, few reports have compared the efficacies of different columns for performing CVVHDF, although several types of columns have been used for this treatment.

PMMA-CVVHDF has been reported to be effective for cytokine removal therapy.[3] Furthermore, our previous paper suggested that PMMA-CVVHDF was more effective than PAN-CVVHDF for the treatment of patients with septic shock, based on the improvements in sepsis-specific factors such as plasminogen activator inhibitor-1 (PAI-1), *N*-arachidonoylethanolamine (AEA), interleukin-6 (IL-6), and protein C.[11] Another paper showed that PMMA-CVVHDF treatment improved both hypercytokinemia assessed by the measurement of the blood IL-6 level and dysoxia assessed by the measurement of the blood lactic acid level.[12] Considering the structural differences between the two columns (PMMA and PAN), the above-mentioned results are not necessarily contradictory. In other words, the PMMA column is a uniform structure with superior adsorbability, whereas the PAN column has a nonuniform structure and superior permeability. Therefore, we suspected that the adsorption of factors such as cytokines might be superior using a PMMA column, compared with a PAN column. Therefore, we think that other columns with uniform structures and adsorbabilities similar to that of the PMMA column might be potentially useful. On the other hand, a recent paper has shown that the use of a single measurement of venous lactic acid, the results of which can be made available soon after admission to the emergency department, provides the clinician with a better risk assessment, possibly enabling a clearer direction to diagnosis and therapy, than a patient’s vital signs.[13] Another paper reported that in patients admitted with clinically suspected infection, the venous lactate level predicted 28-day in-hospital mortality independent of blood pressure and contributed significant prognostic information than that provided by other clinical predictors.[14] Accordingly, we utilized the lactic acid level as a treatment marker for blood apheresis therapy and showed the effectiveness of PMMA-CVVHDF, and compared it with PAN-CVVHDF, based on the improvement in lactic acid. At the same time, we showed that lactic acid was a very important severity marker in sepsis cases and that the lactic acid level was more useful for predicting the outcome of sepsis than common severity scores such as the APACHE II score and the SOFA score. Our study suggests that the PMX column might be effective during the early phase of septic shock before a high level of lactic acid is present.

The limitations of this study included its retrospective design, time frame, and the inclusion of cases in which DHP-PMX treatment was performed under various septic conditions. This is really a “before and after” type of study.

Randomized controlled trial study of CVVHDF showed that early application of standard CVVHF is deleterious in severe sepsis and septic shock.[15] But this study did not use PMMA column.

## Conclusion

Our findings suggest that the PMMA column may be optimal for performing CVVHDF after DHP-PMX treatment, based on the changes in blood lactic acid values.
